# Effect of particle size reduction on the physicochemical and mechanical properties of conventional glass ionomer cement

**DOI:** 10.3389/fdmed.2025.1714410

**Published:** 2025-12-02

**Authors:** Nozimjon Tuygunov, Farangis Abdurahimova, Sevara Rizaeva, Zohaib Khurshid, Arief Cahyanto, Myrna Nurlatifah Zakaria, Bakhtinur Khudanov

**Affiliations:** 1 Faculty of Dentistry, Tashkent State Medical University, Tashkent, Uzbekistan; 2 Department of Restorative Dentistry, Kimyo International University in Tashkent, Tashkent, Uzbekistan; 3 Department of Prosthodontics and Dental Implantology, College of Dentistry, King Faisal University, Al-Ahsa, Saudi Arabia; 4 Center for Artificial Intelligence and Innovation (CAII), Faculty of Dentistry, Chulalongkorn University, Bangkok, Thailand; 5 Department of Clinical Sciences, College of Dentistry, Ajman University, Ajman, United Arab Emirates; 6 Centre of Medical and Bio-allied Health Sciences Research, Ajman University, Ajman, United Arab Emirates

**Keywords:** glass ionomer cement, nano-sized particles, compressive strength, ion release, setting time, pH, dental materials

## Abstract

**Background:**

Conventional Glass Ionomer Cement (GIC) is widely used in restorative dentistry due to its biocompatibility and fluoride release; however, its limited mechanical strength and bioactivity restrict its broader clinical applications. Reducing glass powder particle size represents a promising approach to enhancing its physicochemical performance.

**Objective:**

To investigate the effect of glass powder particle size reduction on the physicochemical and mechanical properties of a conventional GIC.

**Methods:**

Four groups of conventional GIC were prepared by modifying glass powder particle size through one- or two-step ball milling. Particle size distribution (PSD) and field emission scanning electron microscopy (FE-SEM) were used to verify particle morphology, while energy dispersive x-ray spectroscopy (EDX), x-ray diffraction (XRD), and Fourier-transform infrared spectroscopy (FTIR) confirmed chemical composition. The groups included: A – submicron (average 576.9 nm), B – nano (average 92.4 nm), C – hybrid (average 352.6 nm; composed of both nano and submicron particles), and D – control (936.8 nm, unmodified). Evaluations included pH, fluoride, and calcium ion release (over 28 days), initial setting time, compressive strength, and diametral tensile strength. Data were analyzed using one-way analysis of variance (ANOVA) with Tukey's honestly significant difference (HSD) test (*p* < 0.05).

**Results:**

Group B (nano) exhibited the highest fluoride (8.4 ± 0.2 ppm at 3 h) and calcium ion release (1.3 ± 0.08 ppm at 3 h), and the most alkaline pH (6.6 ± 0.09 at day 28). Particle size reduction significantly increased ion release and pH over time but reduced compressive strength (99.02 ± 4.01 MPa) and prolonged setting time (426 ± 10.14 s). The hybrid group (Group C) demonstrated a balanced profile between ion release and mechanical strength, with no chemical alteration observed across groups.

**Conclusion:**

Reducing GIC particle size to the nanoscale enhances ion release and alkalinity but compromises mechanical strength. A hybrid formulation incorporating both nano- and submicron-sized particles provides an optimal balance between bioactivity and strength, offering a promising direction for future development of GICs.

## 1 Introduction

Glass ionomer cements (GICs), introduced by Wilson and Kent over 50 years ago, were initially developed as translucent dental cements. These cements are formulated from two main components: a fluoro-aluminosilicate glass powder and an aqueous solution of polyalkenoic acids, primarily polyacrylic acid. Upon mixing, a neutralization reaction takes place between the acid and basic glass particles, resulting in the formation of water and insoluble polysalt matrices (1, 2). This reaction facilitates the cross-linking of polyacrylate chains with multivalent metal ions such as calcium and aluminum, creating a hardened matrix that encapsulates the glass particles. The final material's mechanical properties—including strength, rigidity, and resistance to solubility—are influenced by the characteristics of its constituents (type of polyacid and glass composition), the powder-to-liquid ratio, and ambient conditions during the setting process (3). GICs are valued in clinical dentistry for their ability to chemically adhere to enamel and dentin, release fluoride ions with anticariogenic effects, and exhibit excellent biocompatibility (4, 5). Owing to these features, GICs are commonly used as luting agents, cavity liners, and restorative materials. Moreover, their compatibility with biological tissues and bone adhesion has extended their applications into neuro-otological and maxillofacial reconstructive surgeries (6, 7).

Despite GICs being widely utilized in restorative dentistry due to their chemical adhesion to enamel and dentin, fluoride release, and biocompatibility, their broader clinical use is hindered by inherent limitations, such as prolonged setting times, suboptimal early mechanical strength, and inconsistent ion-release dynamics (8–11). Efforts to improve these properties have included the incorporation of nano-fillers (12) and changes in material composition; however, most studies have focused on singular modifications—either micro or nano particle formulations—without directly comparing them within a unified GIC matrix (13, 14). The strategic reduction of glass powder particle size is known to enhance packing density, surface reactivity, and microstructural uniformity, potentially improving both mechanical performance and ion-release profiles (13, 15).

Additionally, in restorative dentistry, composite resin materials are categorized based on the size and distribution of their filler particles, with classifications such as macrofilled, microfilled, hybrid, microhybrid, nanofilled, and nanohybrid composites, each tailored to specific clinical applications and performance characteristics (16). This classification system reflects the critical influence of particle size on key material properties such as polishability, wear resistance, and mechanical strength (17). In contrast, GICs, despite being composed primarily of reactive glass powders, are not classified according to particle size (18, 19). Most commercially available GICs are described broadly in terms of viscosity or clinical indication (e.g., conventional, high-viscosity, or resin-modified), with minimal attention to the underlying particle size distribution of the glass component. This lack of granularity may overlook opportunities to tailor GIC properties by manipulating particle size, a strategy that has shown substantial potential in other restorative systems. Nevertheless, the combined effect of submicron, nano, and hybrid (mixed-size) particle distributions on critical functional parameters—such as initial setting kinetics, pH modulation, fluoride and calcium ion release, and long-term material stability—has not been systematically evaluated. This study aimed to investigate the influence of glass powder particle size reduction on the physicochemical and mechanical properties of a conventional GIC, with a particular focus on setting time, pH variation, fluoride and calcium ion release, and compressive strength.

## 2 Methods

### 2.1 Fabrication of GIC powder

A commercially available conventional glass ionomer cement, Fuji IX GP Extra (GC Corporation, Tokyo, Japan), was used in this study to evaluate the effect of particle size reduction on its physicochemical and mechanical properties. According to the manufacturer, Fuji IX GP Extra powder consists of a fluoroaluminosilicate glass containing SiO₂, Al₂O₃, AlF₃, CaF₂, SrO, and P₂O₅, with an average particle size of approximately 8–12 µm prior to any modification. The liquid component is based on an aqueous solution of polyacrylic acid. The GIC powder was subjected to a dry ball milling process using a planetary ball mill (High Energy Ball Mill Emax, Retsch, Germany). In the first milling step, the powder was ground using zirconia balls (1 mm in diameter) at a powder-to-ball weight ratio of 1:10 in a 50 ml zirconia jar at a speed of 300 rpm for 2 h. This one-step milling process produced submicron-sized particles (100–1,000 nm), as confirmed by particle size distribution (PSD) analysis using a Zetasizer Nano (Malvern Panalytical, UK), which were classified as Group A: submicron. To further reduce particle size, the micro-sized powder obtained from the first milling stage was sieved through a 106 µm mesh sieve (Endecotts Ltd., UK). This sieving step was conducted primarily to remove any aggregated or oversized particles and to ensure homogeneity of the feed material for subsequent milling. Particles larger than 106 µm were effectively excluded, thereby defining the upper size limit of the fraction used for nano-milling. The size of the passed particles was subsequently verified by PSD analysis, confirming that all particles were smaller than 106 µm prior to the second milling step. The sieved powder then underwent a second ball milling procedure using smaller zirconia balls (0.5 mm in diameter) under the same conditions (300 rpm for 2 h), resulting in nano-sized particles (1–100 nm), also determined via PSD analysis and validated by FE-SEM imaging (1–100 nm), which were assigned to Group B: nano. The defined particle size ranges for submicron and nano groups were derived from the PSD distribution curves. To prepare Group C: hybrid, micron- and nano-sized powders were mixed in equal weight percentages and uniformly blended using a multipurpose grinder (Bear Electric Appliance Co., Shunde, China) for 1 min to ensure homogeneous distribution under consistent force, speed, and time. The unmodified Fuji IX powder was used as the control in Group D. For all *in vitro* studies, the powder-to-liquid ratio (PLR) was standardized at 3.6:1 (by weight) for all groups, following the manufacturer's recommendation for Fuji IX GP Extra (GC Corporation, Tokyo, Japan). All samples were prepared at room temperature under identical mixing and handling conditions to ensure consistency across the experimental groups.

### 2.2 Sample size calculation

*A priori* sample size calculation was performed using G*Power software (version 3.1) to determine the minimum number of samples required for both physical and mechanical analyses. For ion-release and pH analysis, three independent samples were prepared for each group, and three repeated readings were recorded for each sample at each time interval, resulting in nine independent data points per group per parameter. This approach follows established methodologies reported in previous GIC ion-release studies for pH (20) and F (21), Ca (22) and P ion release (23) assessments and provides adequate statistical power for repeated-measures analysis. For mechanical strength tests (compressive and diametral tensile strength), a minimum of ten samples per group was determined using G*Power analysis to achieve a power of greater than 80% at a significance level of *p* < 0.05 with a 95% confidence interval (24).

### 2.3 Chemical characterization of GIC

#### 2.3.1 FE-SEM and EDX analysis

A field emission scanning electron microscope (FE-SEM) (JEOL JSM-7900F, Tokyo, Japan) with an attached UltiMax 170 mm^2^ Energy dispersive x-ray (EDX) detector (Oxford Instruments, Abingdon, UK) was used to characterize chemical compounds of the prepared samples. Samples from each group were analyzed at 60000X and 120000X magnifications on three separate locations to observe nanoscale features and additives. All samples were analyzed within 24 h of preparation to assess their as-prepared microstructure and elemental composition before any exposure to water or setting reaction. The powders were stored in airtight containers at room temperature (22°C–25°C) and kept in a desiccator under dry air conditions to prevent moisture absorption.

#### 2.3.2 XRD analysis

Characterization of crystalline material was done using x-ray diffractometry (Panalytical X'Pert Pro, Almelo, Netherlands). In this study, the XRD patterns were measured and scanned between 2θ (5°–90°) with a step size of 0.05° in continuous mode at 25°C and a counting time of 2 s per step. The generated data were analyzed using OriginLab software (OriginLab Corp., Northampton, MA, USA), with the use of the JCPDS-ICCD (Joint Committee on Powder Diffraction Standards-International Centre for Diffraction Data) files as references to interpret the XRD patterns provided by each sample. Powder samples were analyzed in their dry, as-prepared state within 24 h of milling, following the same storage protocol as described above.

#### 2.3.4 FTIR analysis

Fourier-transform infrared spectroscopy (Nicolet 6700 FTIR spectrometer, Thermo Fisher Scientific, Waltham, MA, USA) was employed to verify XRD results and identify the structural groups present in the prepared powders. All samples were tested in dry powder form within 24 h after milling, stored in sealed containers at ambient temperature, and protected from moisture prior to analysis. FTIR spectra were recorded in the wavenumber range of 4,000–650 cm⁻^1^ at room temperature and a resolution of 0.5 cm⁻^1^.

### 2.4 Physico-mechanical characterization of GIC

#### 2.4.1 PSD analysis

A particle size analyzer (Zetasizer Nano, Malvern Panalytical, Malvern, UK) was used to measure the particle size distribution (PSD) of the prepared samples. During the process, the measurement of each sample was replicated three times to ensure the reliability and consistency of the results. Thus, the average PSD was calculated using the following equation:

$$\mathrm{Average}\;\mathrm{PSD}\;=\;\frac{\begin{array}{c} \left(\mathrm{average}\;\mathrm{size}\;\mathrm{Peak}\;1*\mathrm{intensity}\;\mathrm{Peak}\;1)+(\mathrm{average}\;\mathrm{size}\;\mathrm{Peak}\;2*\mathrm{intensity}\;\mathrm{Peak}\;2\right)\\ +(\mathrm{average}\;\mathrm{size}\;\mathrm{Peak}\;3*\mathrm{intensity}\;\mathrm{Peak}\;3) \end{array}}{100\text{\%}}$$

#### 2.4.2 Initial setting time

The setting times of each cement (*n* = 3) were measured using a Vicat apparatus (NL Scientific, Selangor, Malaysia) in accordance with ISO 9917 standards (25). The Vicat indenter weighs 400 ± 5 g, with a needle having a flat end diameter of 1.0 ± 0.1 mm. A mold with internal cross-sectional dimensions of 10 × 8 mm^2^ was filled with the prepared cement mixture. Sixty seconds after mixing, the cement samples were incubated in an oven at 37°C. To determine the appropriate setting time, the indentation was repeated at 30 s intervals until the needle failed to make a complete circular indentation in the cement (26).

#### 2.4.3 Fluoride and calcium ions release

To assess F ion release, samples (*n* = 3) of each material were prepared using cylindrical Teflon molds with a diameter of 6 mm and a thickness of 4 mm. The cement was mixed and inserted into the mold for each sample, then covered with a mixing pad on the top and bottom surfaces and compressed. Following an initial setting time of 1 h, each sample was individually immersed in a container with 5 ml of deionized water and stored at 37°C under 100% humidity for 24 h. The F release was measured after 1, 3, 6, 12, and 24 h, as well as after 3, 7, 14, and 28 days, using a pH/ion meter (SevenDirect SD50, Mettler Toledo, China). Prior to analysis, 1 ml of the solution was mixed with 1 ml of TISAB III (Thermo Fisher Scientific, Waltham, MA, USA), and the process was repeated. Following each measurement, the machine was calibrated using F standards (Cole Parmer, Vernon Hills, IL, USA) at concentrations of 0.1, 1, 10, and 100 ppm.

To assess Ca ion release, samples (*n* = 3) of each group were prepared using cylindrical Teflon molds with a diameter of 6 mm and a thickness of 4 mm. The cement was mixed and inserted into the mold for each sample, subsequently covered with a mixing pad on the top and bottom surfaces, and compressed. After an initial setting period of 1 h, each sample was individually immersed in acrylic bottles containing 5 ml of deionized water and stored at 37°C under 100% humidity for 24 h. Calcium ion release was measured using atomic absorption spectroscopy (Perkin Elmer, Waltham, MA, USA). Following the storage period, the calcium concentration was determined by acidifying 1 ml of the sample solution with 0.5 ml of 1 M HCl, followed by dilution with 0.5 ml of 2% lanthanum chloride. The measurements were compared with a calibration curve of seven calcium standards ranging from 0 to 100 ppm. The Ca release of each sample was measured after 1, 3, 6, 12, and 24 h, as well as after 3, 7, 14, and 28 days.

#### 2.4.4 pH Measurement

The pH measurement was conducted in accordance with ISO standards (27). Test materials (*n* = 3) were mixed and placed in a circular Teflon mold with an inner diameter of 6 mm and a height of 4 mm. The molds were then compressed between the mixing pad to remove excess material. After 30 min, the samples were removed from the molds and placed in 100 ml sterile, hermetically sealed polyethylene containers. Five ml of deionized water was added to each container, and the samples were incubated at 37°C. A pH meter (Eutech pH700, Hanoi, Vietnam) was used to measure pH changes by immersing it in the central portion of the solution. The electrode was rinsed between measurements. All pH readings were recorded twice at the following time intervals: 1, 3, 6, 12, and 24 h, as well as 3, 7, 14, and 28 days post-sample preparation. The average pH reading for each sample was then calculated.

#### 2.4.5 Compressive strength analysis

The compressive strength test was conducted in accordance with ISO 9917 requirements (27). Cylindrical samples (*n* = 10) were prepared using PPTF Teflon molds with a measurement of 4 mm and 6 mm in height. The molds were filled with the material, compressed between mixing paper pads, flattened, and gently pressed by hand to remove air bubbles before being clipped. The samples were subsequently incubated at 37°C under 100% humidity for 24 h. A compressive strength test was performed using a universal testing machine (AGS-X Series Shimadzu, Kyoto, Japan) equipped with a 10 kN load cell and a crosshead speed of 10 mm/min. Cylindrical samples (4 ± 0.1 mm in diameter and 6 ± 0.1 mm in height) were prepared following ASTM D695 standards. After being removed from the mold, the dimensions of each sample were verified with a digital caliper (Mitutoyo, Kanagawa, Japan). The compressive strength (C) was calculated in megapascals (MPa) according to the following equation:

$$C\;=\;4P/\pi D^{2}$$

where *P* (N) is the maximum load and *D* (mm) is the diameter of the sample. The test was replicated 10 times for each material (28).

#### 2.4.6 Diametral tensile strength

Ten samples (*n* = 10) for diametral tensile strength (DTS) analysis were prepared using cylinder molds with dimensions 6.0 mm diameter × 3.0 mm height. The molds were filled with the GICs, compressed between mixing paper pads, flattened, and gently pressed by hand to remove air bubbles before being clipped. The samples were subsequently incubated at 37°C under 100% humidity for 24 h. DTS was determined using a universal testing machine (AGS-X Series Shimadzu, Kyoto, Japan) at a crosshead speed of 1.0 mm/min. The maximum load applied to fracture the samples was recorded, and DTS in MPa was calculated using the formula (29):

DTS=2P/πDL

where *P* is the maximum load applied (N), *D* is the measured mean diameter of the sample (mm), and L is the measured length of the sample (mm).

### 2.5 Statistical analysis

Data were recorded and analyzed with SPSS Statistics for Windows version 20.0 (IBM Corp., Armonk, NY, USA). Prior to applying parametric tests, data normality was verified using the Shapiro–Wilk test (for *n* < 50) and the Kolmogorov–Smirnov test (for *n* ≥ 50), while homogeneity of variances was evaluated using Levene's test. The results indicated that all datasets were normally distributed (*p* > 0.05) and satisfied the assumptions for parametric testing. A one-way ANOVA (Analysis of Variance) was performed to evaluate the initial setting time and compressive strength test results. Detection of released ions (F, Ca, P) and pH measurements were measured using repeated measures ANOVA. For *post hoc* analysis, Mauchly's test of sphericity was conducted to determine the appropriate *post hoc* test (Tukey HSD or Dunnett T3) based on the significance level.

## 3 Results

### 3.1 PSD data

**Figure 1:**
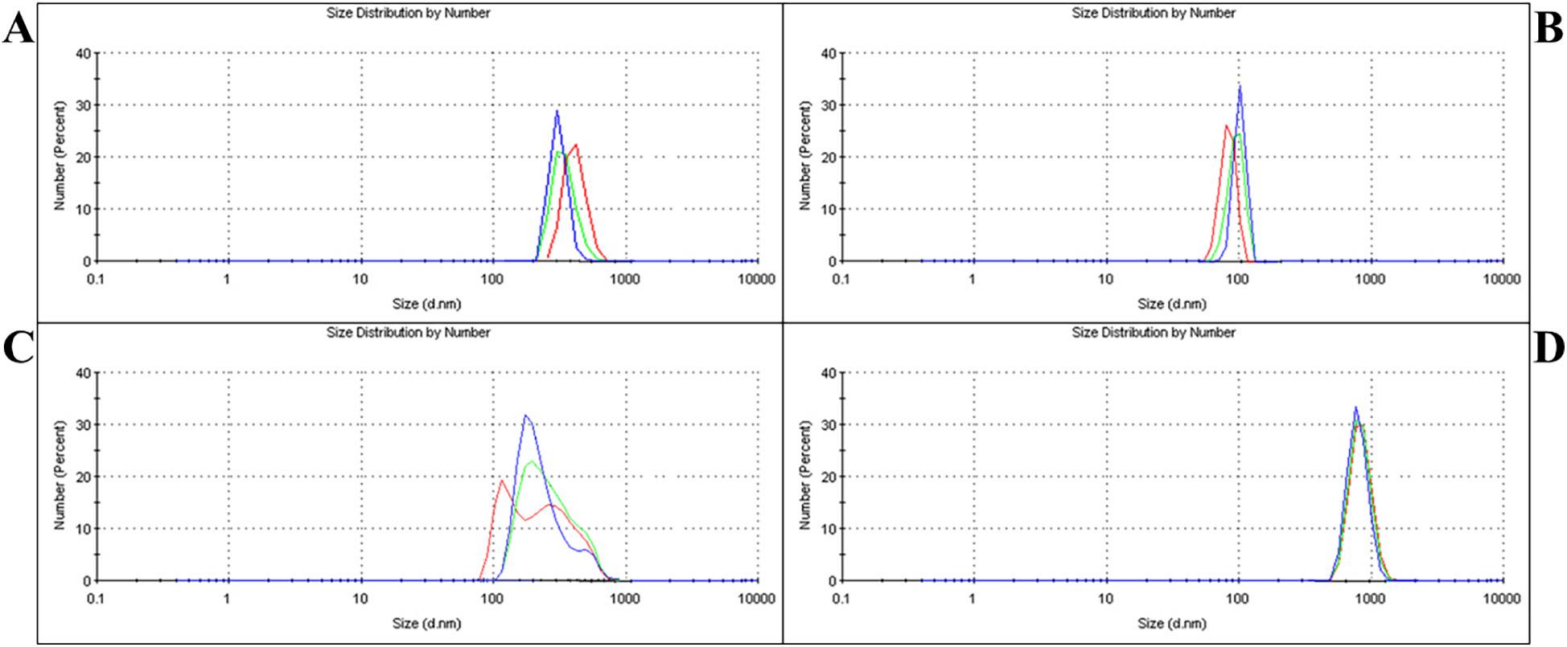
PSD analysis of different groups. **(A)** – Group A: submicron showed an average particle size of 576.9 nm after single-step milling. **(B)** – Group B: nano reached 92.4 nm following two-step milling. **(C)** – Group C: the hybrid had an average size of 352.6 nm, achieved by blending micro- and nano-sized powders. **(D)** – Group D: control exhibited the largest particle size at 936.8 nm.

The PSD of the modified and unmodified GIC powders is presented in Figures 1A–D. Group A: submicron, which underwent a single-step ball milling process, demonstrated an average particle size of 576.9 nm, exhibiting a distribution with 100% homogeneity (Figure 1A). Further particle size reduction via a second ball milling step resulted in Group B: nano with a significantly smaller average PSD of 92.4 nm, also showing 100% homogeneity (Figure 1B). To create a broad-range particle distribution, equal-weight blending of micro- and nano-sized powders produced Group C: hybrid (1–1,000 nm), with an average PSD of 352.6 nm and 100% homogeneity (Figure 1C). In contrast, Group D: control (Unmodified) exhibited the coarsest particle profile, with an average PSD of 936.8 nm, maintaining 100% homogeneity (Figure 1D).

**Figure 2:**
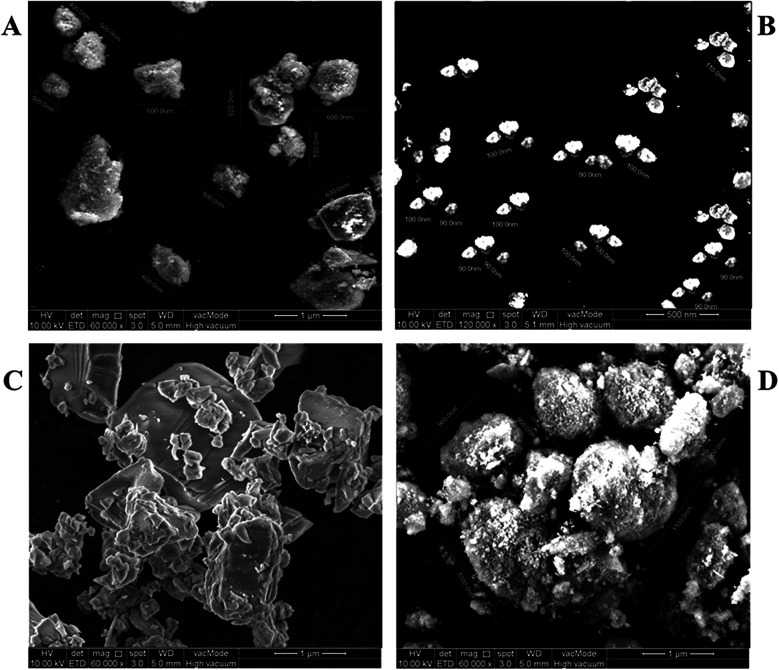
FE-SEM analysis of different groups. **(A)** – Group A: submicron showed an average particle size of 540 ± 66 nm after single-step milling. **(B)** – Group B: nano reached 96 ± 6.2 nm following two-step milling. **(C)** – Group C: hybrid had a blending of submicron and nano-sized powders. **(D)** – Group D: control exhibited the largest particle size at 960 ± 80 nm.

FE-SEM was employed to validate the particle size ranges observed in the PSD analysis. As illustrated in Figures 2A–D, the morphologies and particle sizes observed were consistent with the PSD data. Group A: submicron displayed particles with an average size of approximately 540 ± 66 nm (Figure 2A). In Group B: nano, the particles appeared well-dispersed and nearly spherical, with a measured average size of 96 ± 6.2 nm (Figure 2B). FE-SEM images of Group C: hybrid revealed large micro-sized particles and smaller nano-sized particles embedded on or surrounding the larger surfaces. Due to the inherent complexity of this distribution, precise dimensional measurement was not feasible; however, the images qualitatively confirmed the simultaneous presence of both particle types (Figure 2C). The unmodified powder in Group D: control (Unmodified) exhibited angular, crystalline-like morphologies with an average size of approximately 960 ± 80 nm, consistent with commercial Fuji IX characteristics (Figure 2D).

### 3.2 EDX data

**Figure 3:**
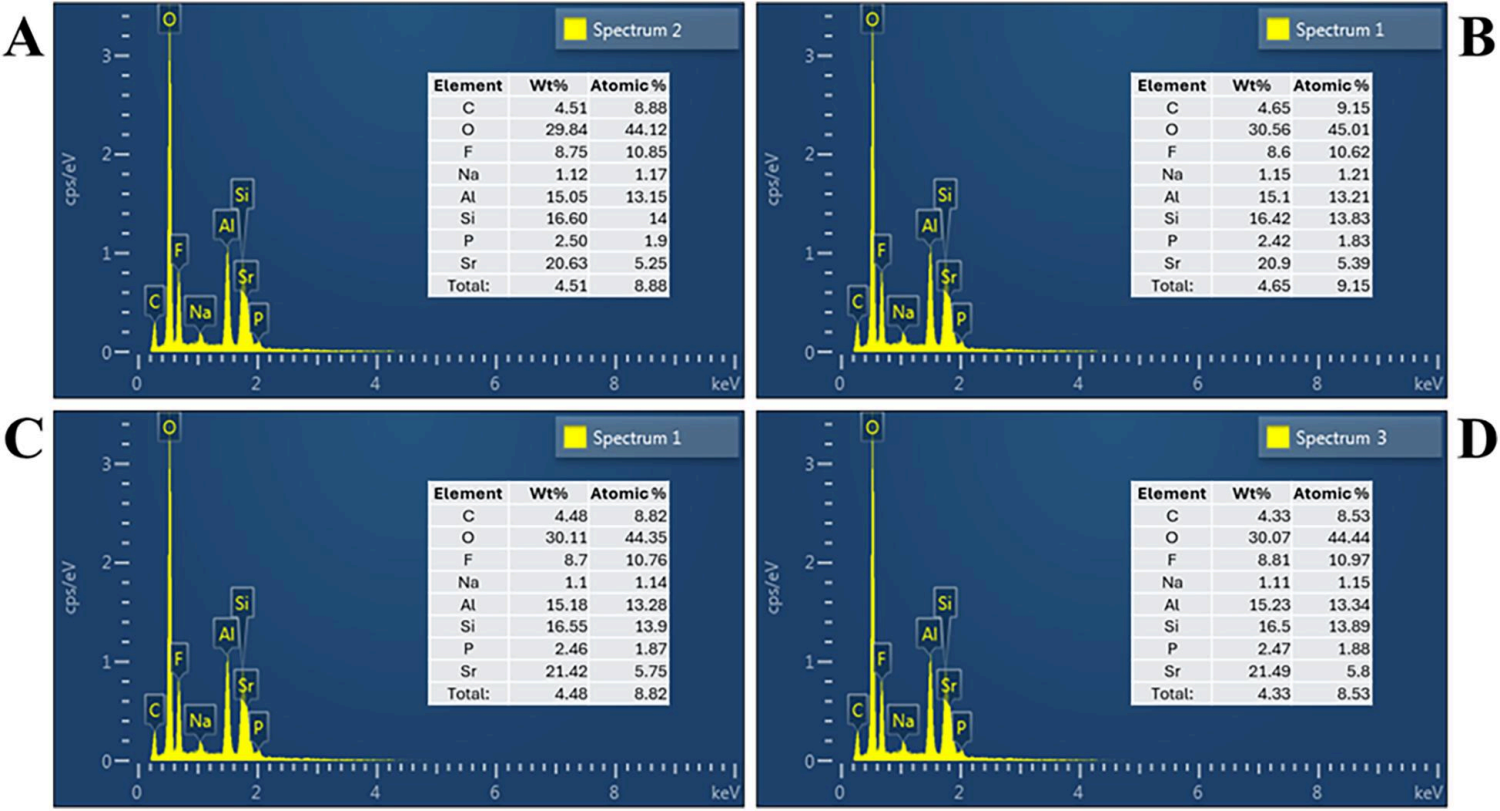
Energy dispersive x-ray spectrum based on respective group: **(A)** group A: submicron, **(B)** group B: nano, **(C)** group C: hybrid, **(D)** group D: control.

The elemental composition of the experimental and control GICs was analyzed using EDX, and the results are presented in Figures 3A–D. All four groups exhibited similar elemental profiles, primarily composed of oxygen (O), silicon (Si), aluminum (Al), fluoride (F), strontium (Sr), phosphorus (P), sodium (Na), and carbon (C), consistent with the expected components of conventional fluoroaluminosilicate-based GICs.

Minor variations in elemental distribution were observed across groups. Oxygen remained the dominant element in all samples, ranging from 29.84 to 30.56 wt% and 44.12 to 45.01 wt%. Fluoride content showed slight differences, with values ranging from 8.6 to 8.81 wt% and 10.62 to 10.97 wt%. Strontium levels, contributing to radiopacity and potential bioactivity, ranged from 20.63 to 21.49 wt% and 5.25 to 5.80 wt%, with the highest detected in Group C (hybrid). Aluminum and silicon, vital for matrix formation and setting, were relatively consistent across groups, ranging from 15.07 to 16.60 wt% for Al and 15.65 to 16.55 wt% for Si.

No significant differences were found in the major elemental compositions among the groups, suggesting that the variation in particle size distribution did not substantially alter the chemical makeup of the cement powder.

### 3.2 XRD and FTIR

**Figure 4:**
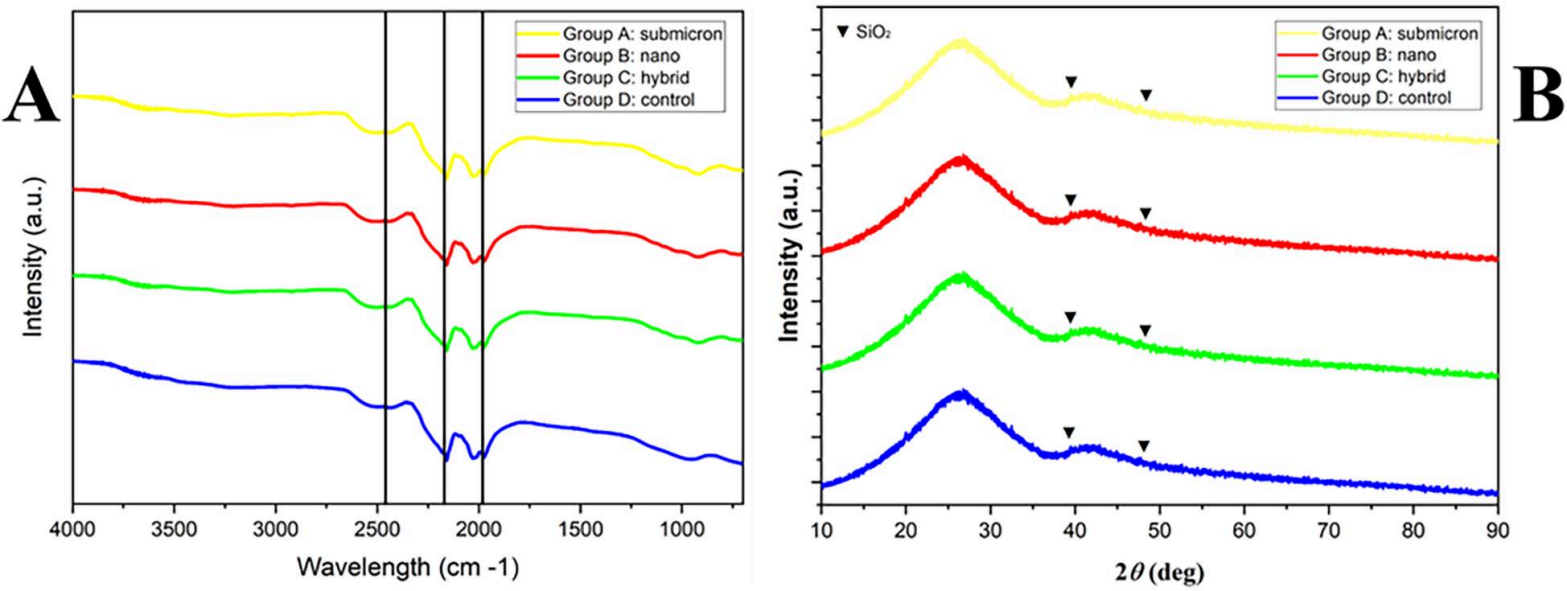
FTIR spectra **(A)** and XRD patterns **(B)** of experimental GIC powders from group A (submicron), group B (nano), group C (hybrid), and group D (control). No significant differences were observed among groups, indicating that the ball milling procedure did not alter the chemical composition or crystallinity of the glass powders.

Figure 4A displays the XRD patterns of all experimental groups, revealing a broad halo between 20° and 40°, indicative of the predominantly amorphous nature of the GIC matrix. Distinct but weak crystalline peaks at 2θ° ≈ 41.9° and 49.5°, corresponding to silicon dioxide (SiO₂) (ICDD 01-075-3169), were observed across all groups. No major shifts or new peaks were detected, suggesting that the ball milling process used to reduce particle size did not significantly affect the crystalline structure.

Figure 4B shows the FTIR spectra of the GIC powders. Characteristic absorption bands were consistently observed in all groups, including Si–O–Si bending vibrations near ∼950 cm^−1^, a Si–C bond around ∼1,980 cm^−1^, and –COO group vibrations at ∼1,980 and ∼2,440 cm^−1^. The spectral similarities across all groups indicate that the chemical composition remained unaltered, confirming that the ball milling procedure did not induce significant chemical modifications in the GIC powders.

### 3.3 Compressive strength and diametral tensile strength

**Figure 5:**
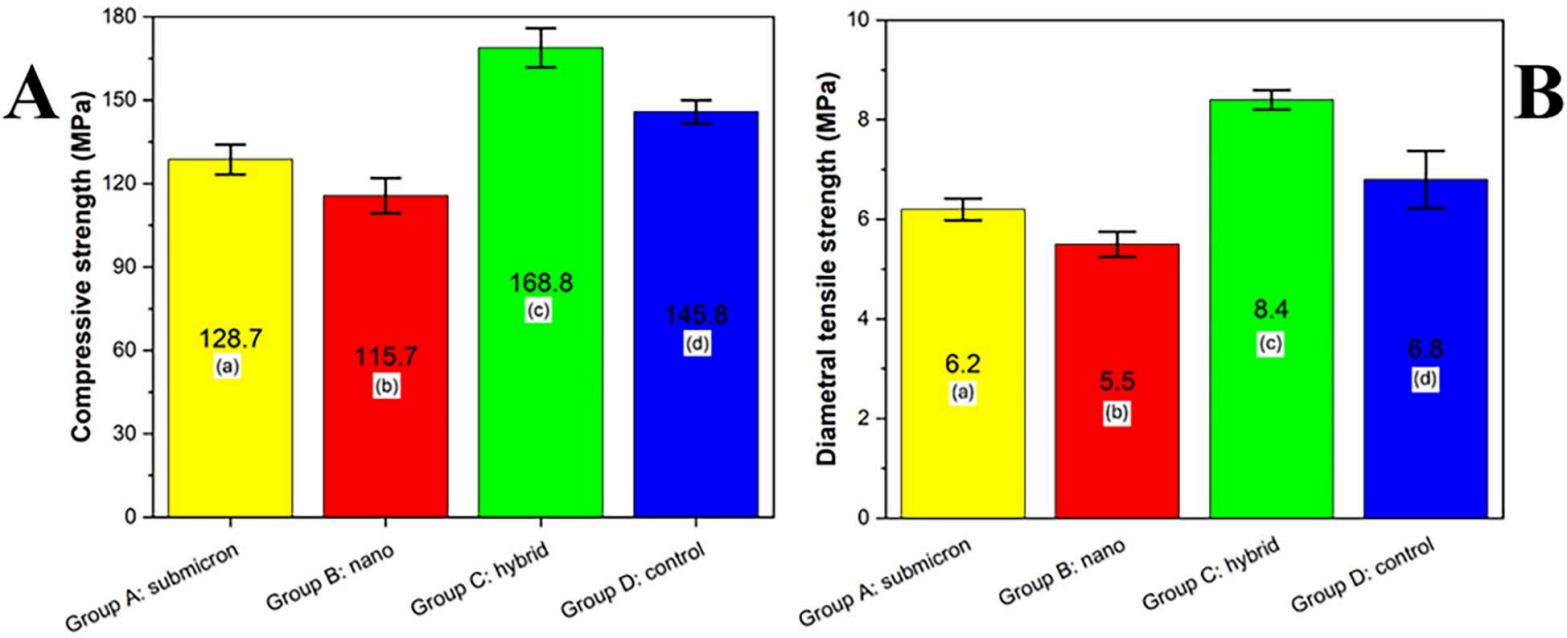
Compressive strength **(A)** and diametral tensile strength **(B)** of experimental GIC groups (MPa) with different particle size distributions. Different lowercase letters indicate statistically significant differences between groups, as determined by Tukey's HSD test (*p* < 0.05).

The results of compressive strength and diametral tensile strength analysis are presented in Figure 5. Among the groups, the hybrid group (Group C) exhibited the highest compressive strength (168.8 MPa), followed by the control group (Group D, 145.8 MPa), submicron group (Group A, 128.7 MPa), and nano group (Group B, 115.7 MPa). Statistical analysis using Tukey's HSD test (*p* < 0.05) revealed that all groups were significantly different from one another, as indicated by the different lowercase letters. These results suggest that particle size modification of the glass powder influenced the mechanical performance of the final GIC, with the hybrid group demonstrating a synergistic enhancement in strength.

The diametral tensile strength (DTS) analysis of the tested groups is presented in Figure 5B. Among the evaluated materials, Group C (hybrid) exhibited the highest DTS value (8.4 MPa), which was statistically significant compared to all other groups (*p* < 0.05). Group D (control) exhibited a moderate DTS of 6.8 MPa, significantly higher than that of Group A (submicron) and Group B (nano), which demonstrated values of 6.2 MPa and 5.5 MPa, respectively. According to Tukey's HSD test, different lowercase letters indicate statistically significant differences among the groups (*p* < 0.05), confirming that the hybrid composition notably enhanced the tensile strength performance compared to the submicron and nano formulations.

### 3.4 Initial setting time

**Figure 6:**
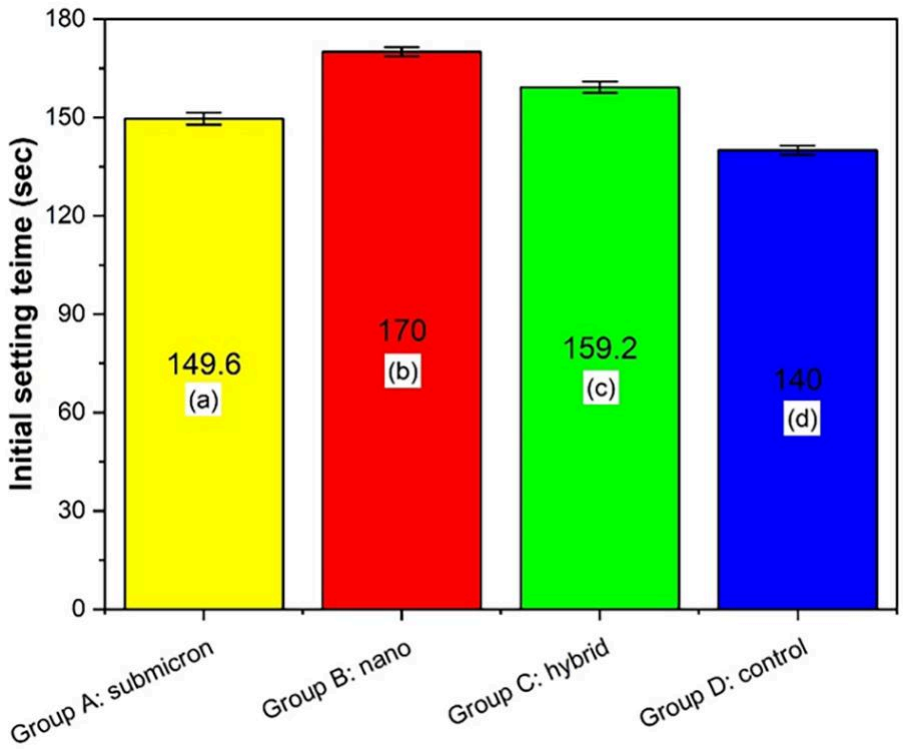
Initial setting time of different groups. Different letters indicate significant differences between the groups, as determined by one-way ANOVA and Tukey HSD *post hoc* testing. (*p* < 0.05).

The initial setting time analysis is presented in Figure 6, revealing statistically significant differences among the groups (*p* < 0.05, Tukey HSD). Group B: nano exhibited the longest setting time of 170 s, which was significantly higher than that of the other groups (*p* < 0.05). This was followed by Group C, which had a setting time of 159.2 s, significantly greater than that of Group A (submicron, 149.6 s) and Group D (control, 140 s). Among all, Group D: control demonstrated the shortest initial setting time. These findings suggest that the particle size of the GIC powder affects the setting kinetics, with nano-sized particles contributing significantly to prolonged setting times, likely due to their increased surface area and altered reactivity during the acid–base reaction.

### 3.5 pH Measurement

**Figure 7:**
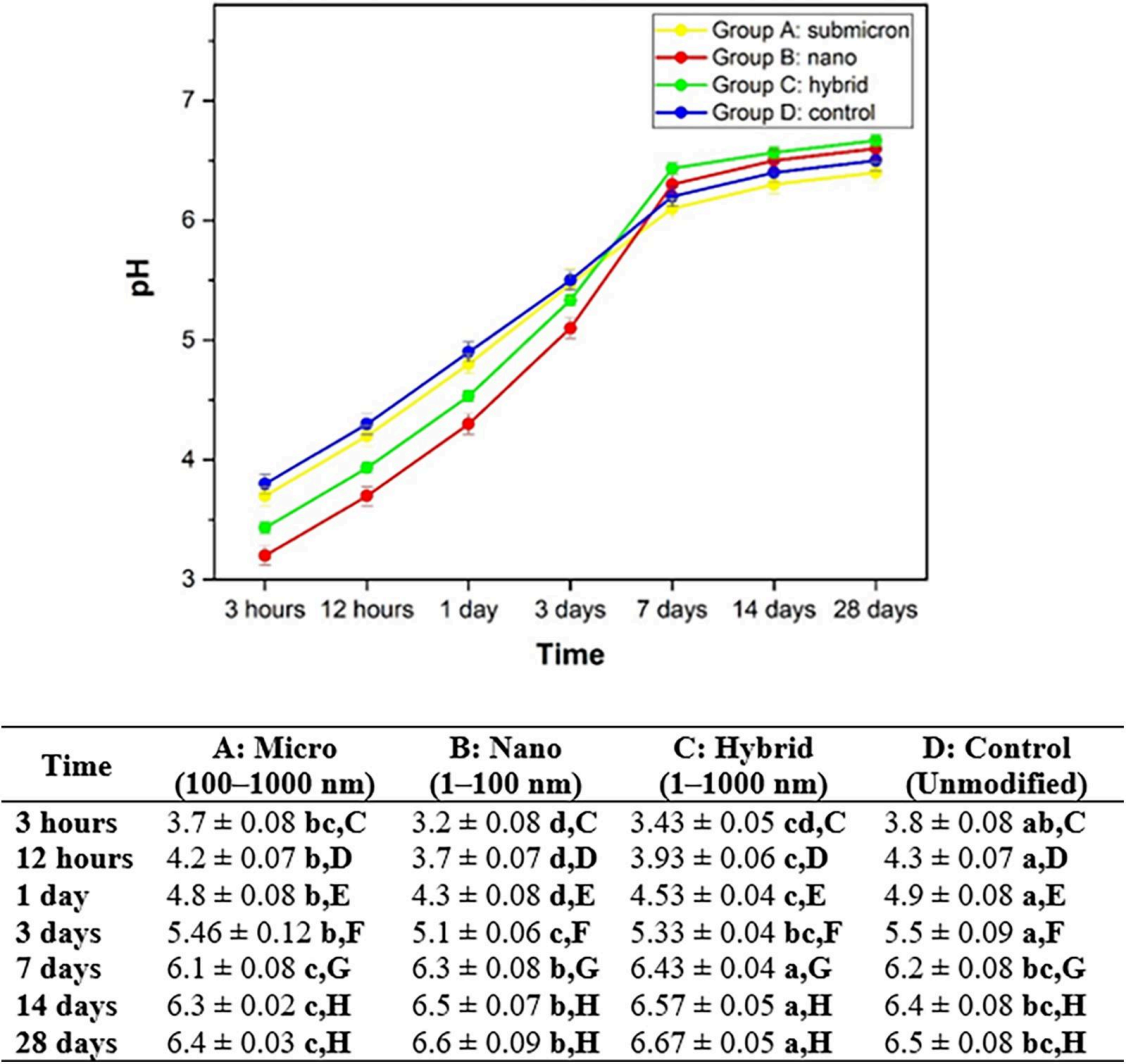
Average and standard deviation of pH after soaking the material in DI water for each set of samples. Different lowercase letters indicate statistically significant differences between different experimental materials in the same measuring period (*P* < 0.05). Different capital letters indicate statistically significant differences between the same experimental material in the different measuring periods (*P* < 0.05).

The pH measurements of GIC cements demonstrated a progressive increase over time, consistent with the typical acid–base reaction and maturation behaviors of these materials. As shown in Figure 7, Group B: nano consistently exhibited the lowest pH values across all time points, starting at 3.2 ± 0.08 at 3 h and increasing to 6.6 ± 0.09 by day 28. In contrast, Group C: hybrid showed the highest pH levels, increasing from 3.43 ± 0.05 at 3 h to 6.67 ± 0.06 at day 28. The submicron-modified group (Group A) demonstrated intermediate pH values ranging from 3.7 ± 0.08 to 6.4 ± 0.03, while the control group (Group D) increased from 3.8 ± 0.08 to 6.5 ± 0.08 over the same period.

Statistical analysis revealed significant differences among the materials at each time point, as indicated by the different lowercase superscript letters (*P* < 0.05). Notably, Group C exhibited significantly higher pH values than Group B from day 1 onward. Within each material group, significant temporal changes were also observed (denoted by different capital letters), especially during the initial 7 days of setting. The nano-sized group demonstrated the slowest rate of pH neutralization, while the hybrid and control groups achieved earlier stabilization. These findings suggest that modifying glass powder with submicron and hybrid particle distributions may enhance the neutralization kinetics of GICs without compromising their long-term stability.

### 3.6 Fluoride and calcium ions release

**Figure 8:**
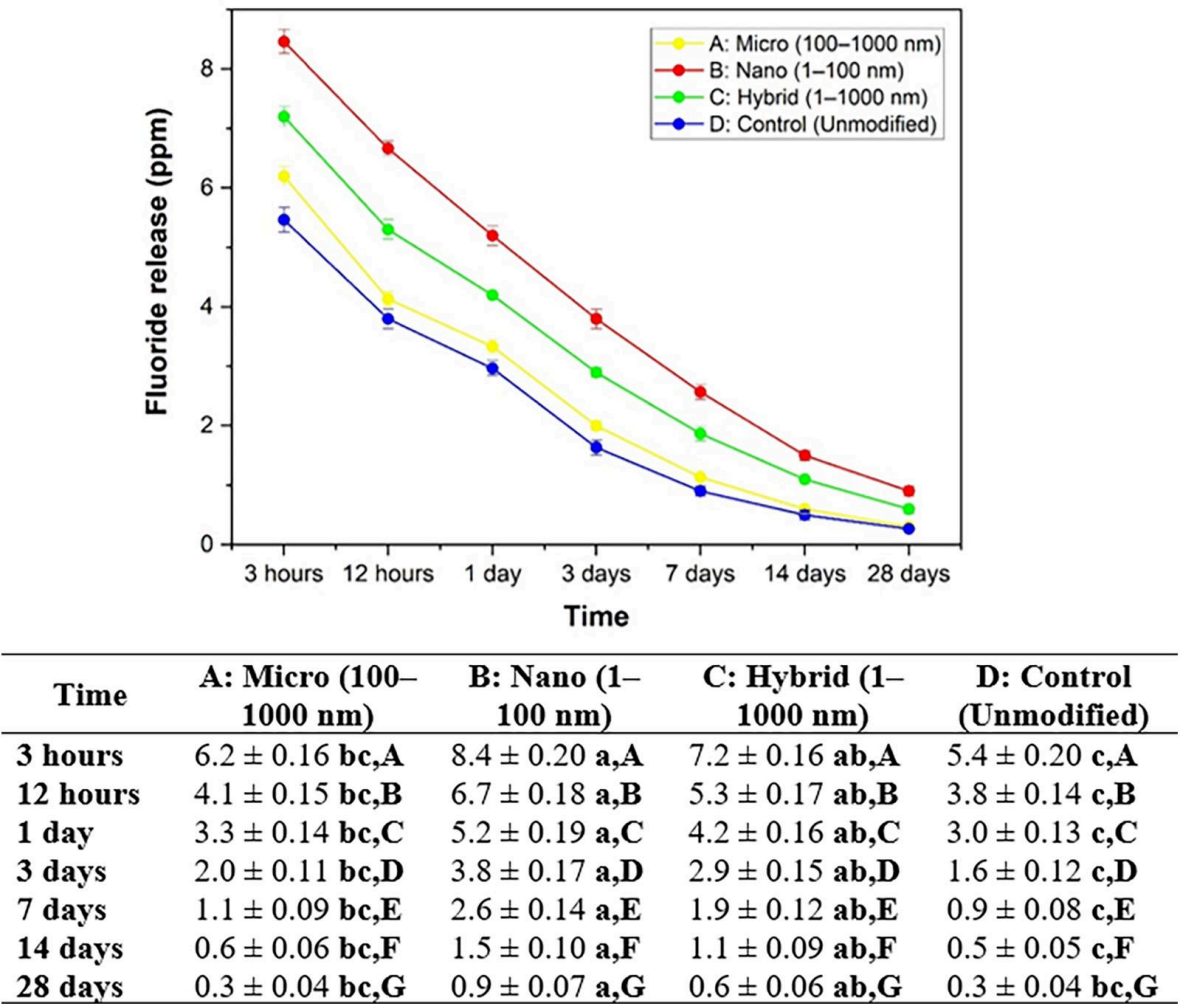
Average and standard deviation of fluoride ion release (ppm) over time after soaking the material in DI water for each set of samples. Different lowercase letters indicate statistically significant differences between different experimental materials in the same measuring period (*P* < 0.05). Different capital letters indicate statistically significant differences between the same experimental material in the different measuring periods (*P* < 0.05).

The fluoride ion release results for all groups at different time intervals are presented in Figure 8. Group B: nano consistently demonstrated the highest fluoride release at all measured time points, with values starting from 8.4 ± 0.20 ppm at 3 h and gradually decreasing to 0.9 ± 0.07 ppm at 28 days. Group C: hybrid exhibited intermediate values, ranging from 7.2 ± 0.16 ppm at 3 h to 0.6 ± 0.06 ppm at 28 days. Group A: submicron released 6.2 ± 0.16 ppm at 3 h, which declined to 0.3 ± 0.04 ppm by day 28. The lowest fluoride release was recorded in Group D: control, from 5.4 ± 0.20 ppm at 3 h to 0.3 ± 0.04 ppm at the end of the study. Statistical analysis revealed significant differences among the groups at each time point (*p* < 0.05), with Group B consistently outperforming the others. Additionally, within each group, fluoride ion release decreased significantly over time, reflecting the expected diffusion-based release behavior following the initial setting phase.

**Figure 9:**
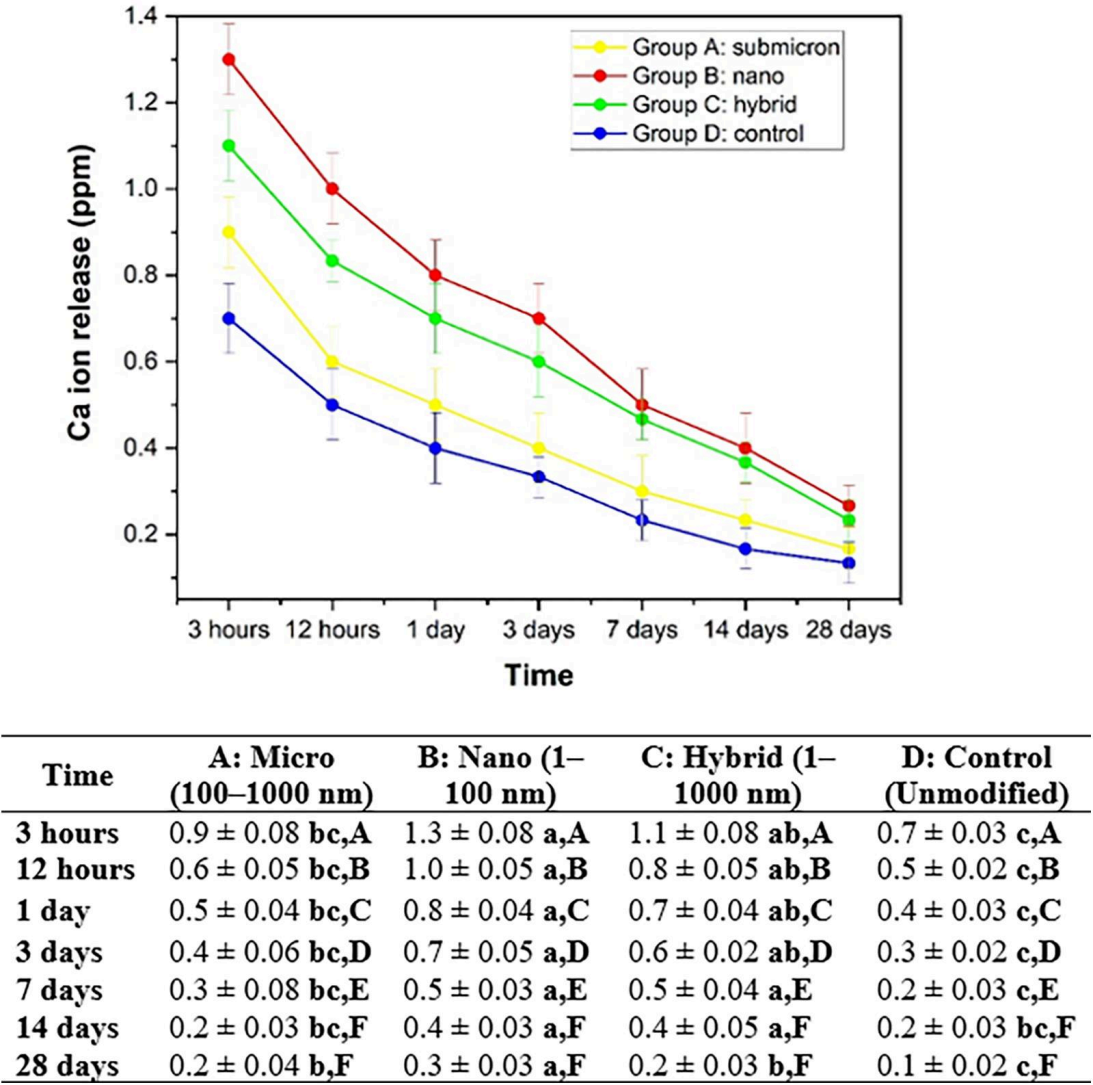
Average and standard deviation of calcium ion release (ppm) over time after soaking the material in DI water for each set of samples. Different lowercase letters indicate statistically significant differences between different experimental materials in the same measuring period (*P* < 0.05). Different capital letters indicate statistically significant differences between the same experimental material in the different measuring periods (*P* < 0.05).

The calcium ion release from all experimental groups gradually decreased over time from 3 h to 28 days (Figure 9). Among all groups, Group B: nano exhibited the highest calcium ion release, beginning at 1.3 ± 0.08 ppm at 3 h and declining to 0.3 ± 0.03 ppm by 28 days. Group C: hybrid followed, releasing calcium from 1.1 ± 0.08 ppm to 0.2 ± 0.03 ppm over the same period. Group A: submicron showed values between 0.9 ± 0.08 ppm and 0.2 ± 0.04 ppm, while Group D: control released the lowest amounts, ranging from 0.7 ± 0.03 ppm at 3 h to 0.1 ± 0.02 ppm at 28 days.

Statistical analysis using Tukey's HSD test (*p* < 0.05) revealed significant differences among the groups at each time point. Lowercase letters indicate differences among the materials within the same time point, while uppercase letters show differences over time within the same material. Group B consistently differed significantly from Group D, particularly at earlier time points (e.g., 3 h and 12 h). The trend suggests that decreasing particle size, especially to the nanoscale, enhances calcium ion release from the GIC matrix, with Group B (nano) showing the most sustained and elevated release.

## 4 Discussion

GICs are not classified by particle size; the variation in the size of the glass powder used is a critical factor influencing their properties and clinical efficacy. A deeper understanding of this aspect can contribute to the development of more specialized and effective GICs tailored for various dental applications. Research by De Caluwé et al. (13) indicated a significant effect of particle size on the properties of GICs, with the potential to enhance their mechanical properties (30).

All GICs exhibited distinct chemical compositions, as confirmed by EDX. While the specific values varied, both GICs were predominantly composed of Al, Si, F, Ca, and P. In the case of Fuji IX GP Fast, as characterized by Yap et al., the EDX analysis revealed that the three primary elements present were oxygen (66.75%), silicon (13.18%), and aluminum (12.82%). Similarly, Fuji IX GP showed the same three main elements in its composition: oxygen (64.42%), silicon (16.77%), and aluminum (16.72%) (31).

**Figure 10:**
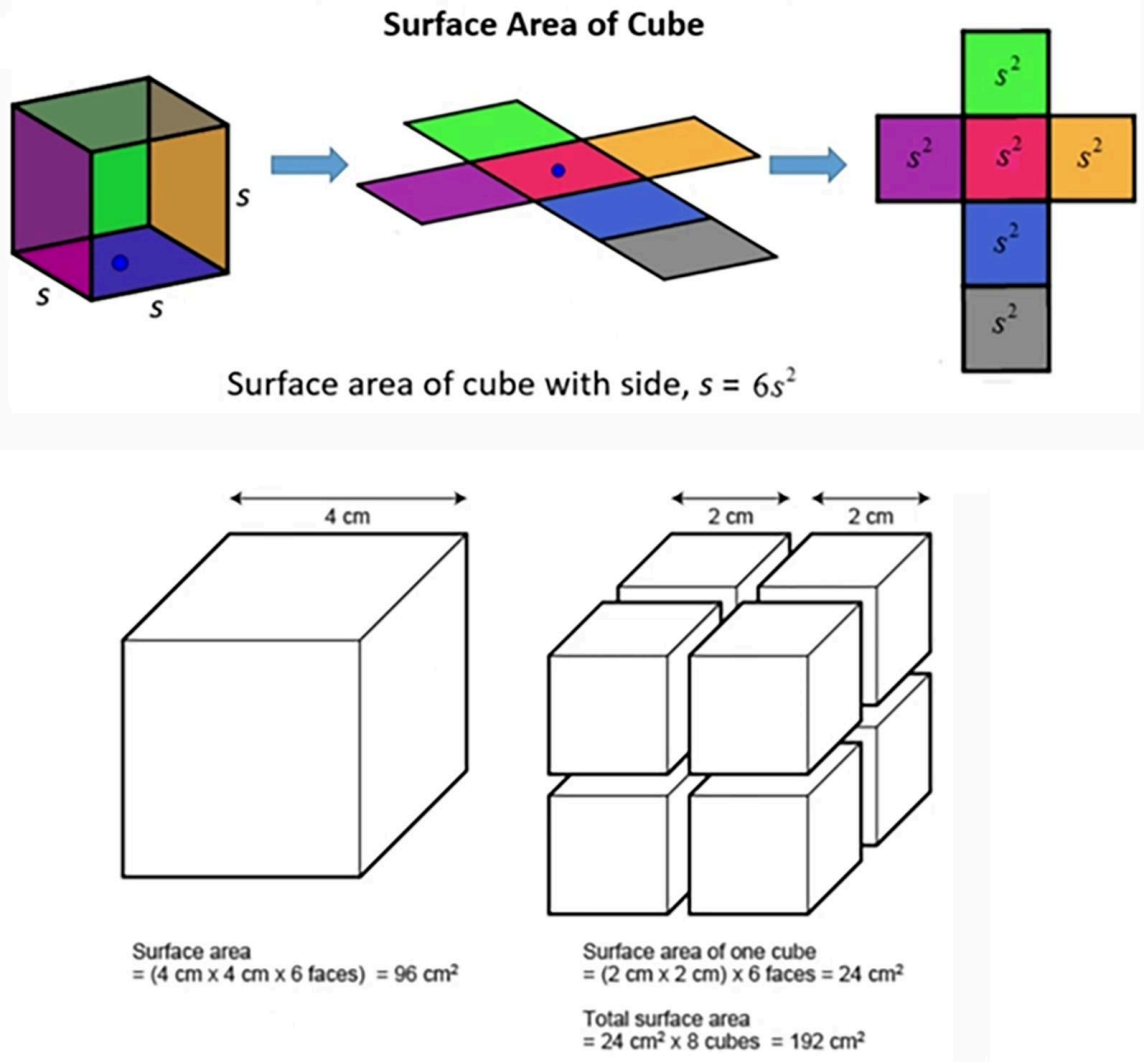
The surface area increases as the particle size decreases. A single 4 cm cube has a total surface area of 96 cm^2^, while eight 2 cm cubes of equal volume yield 192 cm^2^. This illustrates how smaller particles expose more surface, enhancing ion release and reactivity in materials like GIC.

The present study demonstrated that reducing the particle size of GIC powders significantly enhanced the release of fluoride and calcium ions, with the nano-sized group (Group B) exhibiting the highest ion release over time. This phenomenon can be attributed to the fundamental principle that smaller particles lead to higher reactive surface availability, resulting in increased exposure of reactive surfaces to the aqueous environment (32, 33). In materials science and colloid chemistry, it is well-established that as particle size decreases, the total surface area available for ion exchange, diffusion, and reactivity increases exponentially (34, 35). For example, when a cube is divided into smaller cubes, the total surface area increases significantly, thereby enhancing the interaction between the particles and the surrounding fluid (Figure 10).

In the context of GICs, this increased surface area facilitates greater acid-base reactions between the polyacrylic acid and the glass particles, leading to more pronounced ion exchange and leaching of bioactive ions (8, 23). Similar findings were reported by Moshaverinia et al., who showed that GICs containing nano-sized fillers exhibited superior fluoride and calcium ion release compared to conventional formulations (15). Additionally, Barandehfard et al. observed that nano-hydroxyapatite-modified GICs demonstrated increased bioactivity and ion diffusion due to higher surface exposure (36, 37). These outcomes are consistent with our current results, supporting the hypothesis that particle size reduction enhances the physicochemical performance of GICs by increasing the availability of ion-releasing surfaces. On the other hand, the subsequent sustained release of fluoride and calcium ions is primarily attributed to the gradual dissolution of unreacted or partially reacted glass particles entrapped within the polysalt matrix, rather than to continued acid–base reactivity (38).

Furthermore, the enhanced ion release observed in our study may offer improved clinical benefits, such as better remineralization potential and cariostatic effect, particularly when nano-modification is employed. However, it is noteworthy that the hybrid group (Group C), which contains a blend of nano and submicron particles, also exhibits favorable ion release while maintaining optimal mechanical properties, suggesting a balance between surface reactivity and structural integrity (39, 40).

In addition to ion release, the pH behavior of all groups demonstrated a progressive increase in pH values over time, which is consistent with the characteristic neutralization of acidity during the maturation of glass ionomer cements. Among the groups, Group B: nano exhibited the most pronounced initial acidity (pH 3.2 ± 0.08 at 3 h), which gradually increased to a more neutral value of 6.6 ± 0.09 at 28 days. This trend is reflective of the enhanced acid-base reactivity associated with higher surface area in nano-sized particles, leading to more rapid and extensive dissolution of ion-leachable components. In contrast, Group D: control exhibited relatively higher initial pH (3.8 ± 0.08), indicating a slower or less intense reaction process, likely due to reduced surface availability for polyacid attack (13, 41). These findings align with previous studies, which have reported that nano-modified GICs initially demonstrate a lower pH but stabilize more quickly during maturation. The transient low pH phase is essential for the initial setting reaction, while the gradual rise supports long-term biocompatibility and the potential for enamel remineralization in clinical use (1, 15).

The observed variations in compressive strength among the experimental groups highlight the impact of particle size distribution on the mechanical behavior of GICs. The significantly higher compressive strength in Group C: hybrid (168.8 ± 4.7 MPa), compared to the control group (145.8 ± 4.3 MPa), suggests that incorporating both nano- and submicron-sized particles may enhance packing efficiency, reduce microvoids, and improve the structural integrity of the set cement. This finding is consistent with previous studies, which have shown that bimodal particle distributions enhance the density and strength of restorative materials by improving particle packing and reducing interstitial space (42).

While the nano group (Group B) exhibited the smallest particle size, its compressive strength was the lowest among all groups (115.7 ± 3.9 MPa). This may be attributed to particle agglomeration, a phenomenon commonly discussed in the context of nanoscale materials. Agglomerated particles can act as stress concentrators, disrupting the homogeneity of the matrix and ultimately compromising mechanical strength, as well as increasing the fundamental surface area per unit mass (9). As particles become smaller, their surface-to-volume ratio increases substantially, and correspondingly, more surface energy is required to achieve interparticle bonding and matrix cohesion. This elevated energy demand may hinder complete bonding during the acid-base setting reaction, resulting in weaker mechanical properties. Similar findings have been reported in nanofilled resin-based and cementitious materials, where excessive surface energy impedes densification and compromises strength (13, 43). The superior compressive strength observed in Group C: hybrid (168.8 ± 4.7 MPa) highlights the benefit of incorporating both nano- and submicron-sized particles to optimize particle packing while mitigating the drawbacks of nanoscale surface energy imbalance.

The observed variation in initial setting times among the groups is consistent with the notion that particle size can significantly influence the kinetics of the acid-base reaction in GICs. In this study, Group B: nano demonstrated a noticeably prolonged setting time (7.4 ± 0.15 min) compared to Group A: submicron (5.2 ± 0.18 min) and Group D: control (4.9 ± 0.17 min). This delay may be attributed to the increased surface area of nano-sized particles. Previous studies have indicated that finer particles, despite their reactive potential, can exhibit slower setting due to their tendency to form more viscous slurries, which limits ion mobility during the early stages of setting (9, 44). Furthermore, excess surface area may adsorb more polyacid, delaying the neutralization reaction necessary for gelation and hardening (45). Interestingly, Group C: hybrid, which combined nano and submicron particles, achieved a moderate setting time (6.1 ± 0.13 min), suggesting a more balanced reaction kinetics likely due to optimized particle packing and an improved acid-base reaction interface. This aligns with the findings by Nicholson et al., who emphasized that ideal setting performance in GICs requires not only particle fineness but also appropriate distribution and reactivity to ensure cohesive matrix formation (8). Although this sample size meets the calculated statistical requirements, the limited number of samples for ion-release and pH analysis is acknowledged as a study limitation. Future investigations should consider increasing the number of samples and further improving the reliability and reproducibility of the results.

## 5 Conclusion

This study demonstrated that particle size modification of GIC powders significantly affects their physicochemical and mechanical properties. The nano-sized GIC group exhibited enhanced ion release behavior, including increased fluoride and calcium ion release, as well as elevated pH over time, which may contribute to improved bioactivity and remineralization potential. These improvements are likely due to the greater surface area of nanoparticles, which facilitates faster and more extensive ion exchange. However, the reduction in particle size to the nano scale was accompanied by a noticeable decline in compressive strength and a prolongation of the initial setting time, possibly due to weaker interparticle bonding and delayed matrix formation. Conversely, the hybrid group, which combined nano and submicron particles, showed a favorable balance between mechanical integrity and ion-releasing capability, suggesting that a bimodal particle distribution may optimize both strength and bioactivity. Importantly, EDX, XRD, and FTIR analyses confirmed that the ball milling process did not alter the chemical composition of the glass particles. Collectively, these findings suggest that tailoring GIC particle size offers a promising strategy to enhance its performance, but a balance between reactivity and structural integrity must be carefully managed.

## Data Availability

The original contributions presented in the study are included in the article. Further inquiries can be directed to the corresponding author.
