# Correction: Effect of particle size reduction on the physicochemical and mechanical properties of conventional glass ionomer cement

**DOI:** 10.3389/fdmed.2026.1914346

**Published:** 2026-07-09

**Authors:** Nozimjon Tuygunov, Farangis Abdurahimova, Sevara Rizaeva, Zohaib Khurshid, Arief Cahyanto, Myrna Nurlatifah Zakaria, Bakhtinur Khudanov

**Affiliations:** 1 Faculty of Dentistry, Tashkent State Medical University, Tashkent, Uzbekistan; 2 Department of Restorative Dentistry, Kimyo International University in Tashkent, Tashkent, Uzbekistan; 3 Department of Prosthodontics and Dental Implantology, College of Dentistry, King Faisal University, Al-Ahsa, Saudi Arabia; 4 Center for Artificial Intelligence and Innovation (CAII), Faculty of Dentistry, Chulalongkorn University, Bangkok, Thailand; 5 Department of Clinical Sciences, College of Dentistry, Ajman University, Ajman, United Arab Emirates; 6 Centre of Medical and Bio-allied Health Sciences Research, Ajman University, Ajman, United Arab Emirates

**Keywords:** glass ionomer cement, nano-sized particles, compressive strength, ion release, setting time, pH, dental materials

In the published article, there was an error in the Results section, 3.3 Compressive Strength and Diametral Tensile Strength. “The text incorrectly referred to ‘Figure X’. The correct figure reference is ‘Figure 5B’.”

The sentence was originally published as:

“The diametral tensile strength (DTS) analysis of the tested groups is presented in Figure X.”

A correction has been made to the section Results, 3.3 Compressive Strength and Diametral Tensile Strength, Paragraph 2:

“The diametral tensile strength (DTS) analysis of the tested groups is presented in Figure 5B. Among the evaluated materials, Group C (hybrid) exhibited the highest DTS value (8.4 MPa), which was statistically significant compared to all other groups (*p* < 0.05). Group D (control) exhibited a moderate DTS of 6.8 MPa, significantly higher than that of Group A (submicron) and Group B (nano), which demonstrated values of 6.2 MPa and 5.5 MPa, respectively. According to Tukey's HSD test, different lowercase letters indicate statistically significant differences among the groups (*p* < 0.05), confirming that the hybrid composition notably enhanced the tensile strength performance compared to the submicron and nano formulations.”

Author Bakhtinur Khudanov was erroneously assigned to affiliation “7. Faculty of Dentistry, Impulse Medical Institute, Tashkent Region, Uzbekistan”. This affiliation has now been removed from the article.

The original version of this article has been updated.

